# Nanoparticles Suitable for BCAA Isolation Can Serve for Use in Magnetic Lipoplex-Based Delivery System for L, I, V, or R-rich Antimicrobial Peptides

**DOI:** 10.3390/ma9040260

**Published:** 2016-03-31

**Authors:** Radek Vesely, Pavlina Jelinkova, Dagmar Hegerova, Natalia Cernei, Pavel Kopel, Amitava Moulick, Lukas Richtera, Zbynek Heger, Vojtech Adam, Ondrej Zitka

**Affiliations:** 1 Department of Traumatology at the Medical Faculty, Masaryk University and Trauma Hospital of Brno, Ponavka 6, Brno CZ-662 50, Czech Republic; r.vesely@unbr.cz; 2 Department of Chemistry and Biochemistry, Mendel University in Brno, Zemedelska 1, Brno CZ-613 00, Czech Republic; jelinkova.pav@gmail.com (P.J.); dagmar.chudobova@centrum.cz (D.H.); cernei.natalia3@gmail.com (N.C.); paulko@centrum.cz (P.K.); amitavamoulick@gmail.com (A.M.); oliver@centrum.cz (L.R.); heger@mendelu.cz (Z.H.); vojtech.adam@mendelu.cz (V.A.); 3 Central European Institute of Technology, Brno, University of Technology, Technicka 3058/10, Brno CZ-616 00, Czech Republic

**Keywords:** branched chain amino acids, encapsulation, *Escherichia coli*, nanomedicine, *Staphylococcus aureus*

## Abstract

This paper investigates the synthesis of paramagnetic nanoparticles, which are able to bind branched chain amino acids (BCAAs)—leucine, valine, and isoleucine and, thus, serve as a tool for their isolation. Further, by this, we present an approach for encapsulation of nanoparticles into a liposome cavity resulting in a delivery system. Analyses of valine and leucine in entire complex show that 31.3% and 32.6% recoveries are reached for those amino acids. Evaluation of results shows that the success rate of delivery in *Escherichia coli* (*E. coli*) is higher in the case of BCAAs on nanoparticles entrapped in liposomes (28.7% and 34.7% for valine and leucine, respectively) when compared to nanoparticles with no liposomal envelope (18.3% and 13.7% for valine and leucine, respectively). The nanoparticles with no liposomal envelope exhibit the negative zeta potential (−9.1 ± 0.3 mV); however, their encapsulation results in a shift into positive values (range of 28.9 ± 0.4 to 33.1 ± 0.5 mV). Thus, electrostatic interactions with negatively-charged cell membranes (approx. −50 mV in the case of *E. coli*) leads to a better uptake of cargo. Our delivery system was finally tested with the leucine-rich antimicrobial peptide (FALALKALKKALKKLKKALKKAL) and it is shown that hemocompatibility (7.5%) and antimicrobial activity of the entire complex against *E. coli*, *Staphylococcus aureus* (*S. aureus*), and methicilin-resistant *S. aureus* (MRSA) is comparable or better than conventional penicillin antibiotics.

## 1. Introduction

The multidisciplinary field of nanotechnologies has engulfed a myriad of new opportunities concerning areas of interest like health care, material sciences, or analytical chemistry [1]. Nanomaterials-based isolation and preconcentration play important roles in many analytical procedures, such as the elimination of interfering substances [2,3]. Moreover, the advantage of nanomaterial utilization is possibility of their modification with various chemical groups to increase their selectivity [4,5]. The versatility of paramagnetic particles challenges to expand possibilities of their utilization and develop new combinations, improving their excellent qualities [6]. Paramagnetic particles (PMPs) are of great interest due to their unique purposes. Especially in medicine, their application is quite promising. PMPs have been actively investigated as the next generation of targeted drug delivery for more than thirty years. The importance of targeted drug delivery is to transport a drug directly to the center of the disease under various conditions and, thereby, treat it deliberately and/or to enhance the interactions between delivery system and the selected target (pathogen, cancer cells, *etc.*) [7].

Hence, the present work is focused on the synthesis of nanomaghemite particles and their modification using (3-aminopropyl) triethoxysilane (APTES), which will be able to establish a bond with branched chain amino acids (BCAAs). BCAAs are essential in many processes in mammalian bodies. They act as important activators of mammalian targets of rapamycin (mTOR) signaling cascade [8], involved, for example, in cardiac hypertrophy in regulation of proteosynthesis [9]. The depletion of BCAAs may result in alterations in mTOR functions, subsequently influencing protein synthesis [10]. Therefore, nanoparticles for isolation and/or preconcentration of BCAAs from bodily fluids may be applicable as magnetically-immobilizable recognition elements in biosensors. Furthermore, BCAAs are often utilized as a bioactive part of cationic peptides with antimicrobial activity (mostly leucine-rich and valine-rich repetitions, possessing high antibacterial activity and good hemocompatibility) [11,12]. Nanoparticles for their delivery can, thus, serve as a platform for further specific modifications leading to decrease of their non-target toxicity.

The second goal of our study was the enclosure of the PMPs with bound amino acids into a cationic liposome. Magnetic liposomes—phospholipid vesicle bearing magnetic or paramagnetic nanoparticles—were previously suggested as contrast agents for magnetic resonance imaging (MRI), with a possibility to be functionalized simply for ligand-specific targeting [13,14]. Moreover, a targeting of various antimicrobial agents by means of liposomes is in the forefront of the research of the treatment of infections that prove refractory to conventional forms of antimicrobial agents, such as amphotericin B, nystatin, or macrolide [15,16]. Liposomal encapsulation also offers an efficient way of delivery of antimicrobial peptides, as has been shown by Yang *et al.* on a methicillin-resistant *Staphylococcus aureus* (MRSA) [17]. Thus, we hypothesized that the cationic nature of the complexes formed by PMPs and cationic liposomes may improve the interactions with the bacterial membranes, and, hence, it may be useful in bacterial infection treatment [18]. It has been shown that our nanostructure provides response to external magnetic fields and better bioavailability, which was proved by an *in vitro* experiment using an *E. coli* strain, tested for uptake of BCAAs from nanoparticles. Undoubtedly, there is an urgent need for the development of new effective antimicrobial modalities to fight against accelerating bacteria multi-drug resistance by natural evolution. Hence, we finally employed nanoparticles for immobilization of cationic leucine-rich antimicrobial peptides. Consequent testing on *E. coli*, *S. aureus*, and MRSA strains revealed significantly higher antimicrobial activity of liposomal formulation compared to naked peptides. We have also decided to compare the efficiency of our designated system with the antimicrobial effects of the conventional antibiotic drug—penicillin. The rationality of selection was that the penicillin class of drugs still remains a highly valuable group of antibiotics in primary care. Emergence of resistant bacterial strains has limited the usefulness of penicillins in recent years. However, penicillins remain the drugs of choice for many mild, localized, soft-tissue infections [19]. Overall, our delivery system can be useful for the delivery of BCAA-rich antimicrobial peptides. The liposomal envelope protects the cargo against unwanted interactions with the environment and can be further modified with a broad spectrum of active biomolecules.

## 2. Results and Discussion

### 2.1. Characterization of Nanomaghemite@APTES Conjugate’s Physicochemical Properties

**Figure 1:**
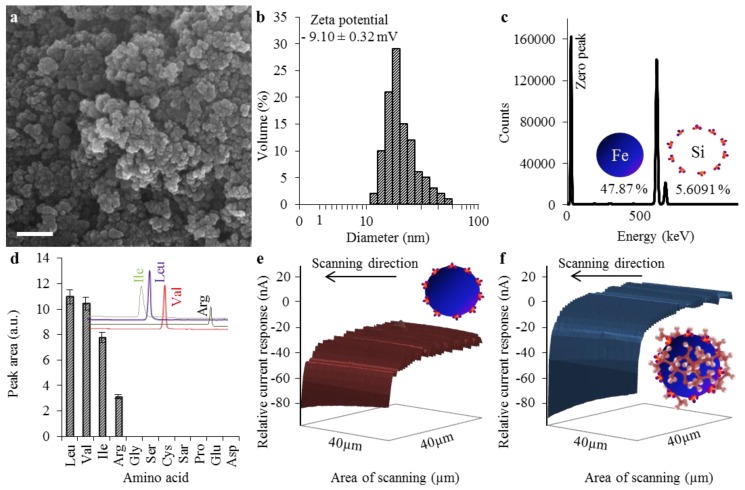
Paramagnetic particles (PMPs) composed of a nanomaghemite core, covered with (3-aminopropyl) triethoxysilane (APTES), were characterized to obtain an insight into their morphology, expressed as: (**a**) SEM micrograph (the length of scale bar is 100 nm); (**b**) size distribution of paramagnetic particles (NaCl solution with pH 10), with expression of zeta potential, measured in aqueous solution (pH = 7); (**c**) XRF elemental analysis. The zero peak serves as a control; (**d**) ion-exchange liquid chromatography (IEC) expression of the specificity of chemical moieties. Chromatograms of the most abundant analytes are shown to represent the differences in retention times. IEC experiments used standards in concentrations of 100 µg·mL^−1^; (**e**) a SECM scan represents the relative current response of paramagnetic microparticles without analyte; and (**f**) a SECM scan of a paramagnetic microparticle with leucine bound showing increased relative current response caused by APTES protonation.

Scanning electron microscopy (SEM) is an efficient way for characterization of the surfaces of various materials [20]; hence, it was used to examine the aspect of paramagnetic particles’ behavior. In Figure 1a (scale bar of 100 nm), spherical-shaped nanoparticles in agglomeration, caused by relatively low zeta potential, established to −9.10 ± 0.32 mV (mentioned in Figure 1b) are shown. Low zeta potential results in a low degree of repulsion between nanoparticles and, thus, stability of ferrofluid cannot be conferred and aggregation occurs [21]. These data correspond to a general statement that iron oxide nanoparticles exhibit absolute zeta potential (indicator for a stable dispersion) above pH 10 and beneath pH 4 [22]. The nanoparticle suspension consisted of primary particles with relatively uniform size (d = 20 nm), when measured in NaCl solution with pH 10 (Figure 1b). The nanoparticles agglomerated to aggregates of a mean size of d = 100 nm when determined in aqueous solution, as can be seen in the SEM micrograph in Figure 1a. The next characterization step included a collection of X-ray fluorescence (XRF) spectra, providing basic information about nanoparticles composition. As it can be seen in Figure 1c, iron (Fe) was identified as the most abundant element (47.9%), originated from utilization of nanomaghemite (γ-Fe_2_O_3_) to constitute the core of a nanoparticle. Silicon (Si) was determined as the second most abundant element (5.6%) and originated from APTES, forming a shell of a nanostructure and providing chemical moieties towards binding to the analyzed substance.

To explore the specificity of chemical moieties of the nanoconjugate, ion-exchange liquid chromatography (IEC) was employed. Samples were prepared as described in Section 3. It was determined that the paramagnetic nanoparticles were suitable for isolation of leucine (Leu, peak area 10.97 mAU), valine (Val, peak area 10.43 mAU), isoleucine (Ile, peak area 7.76 mAU, belonging to the group of BCAAs), and arginine (Arg, peak area 3.14 mAU). Other tested amino acids were shown to have no willingness to establish bonding with particles prepared by us (Figure 1d).

Finally, we included scanning electrochemical microscopy (SECM) to obtain the insight into paramagnetic nanoparticles surface relative current response before and after binding of amino acids. A scan of the layer comprising bare nanoparticles (40 µm × 40 µm) revealed relative current response of approximately −20 nA (Figure 1e). It was shown that the isolation procedure leads to changes in the nanoparticles’ electrochemical behavior. APTES (pI 4 [23]) aminopropyl groups were protonated, *i.e*., positively-charged, due to washing with Britton-Robinson buffer (pH 2); hence, the relative current response of nanoparticles was increased to approx. 19 nA (Figure 1f). Protonation of APTES moieties leads to introduction of α-ammonium groups for binding leu, which forms a zwitterionic structure (pKa 2.36 and 9.60 for carboxyl and amino moieties, respectively) in neutral pH (7.4), maintained by phosphate buffer saline [24].

**Figure 2:**
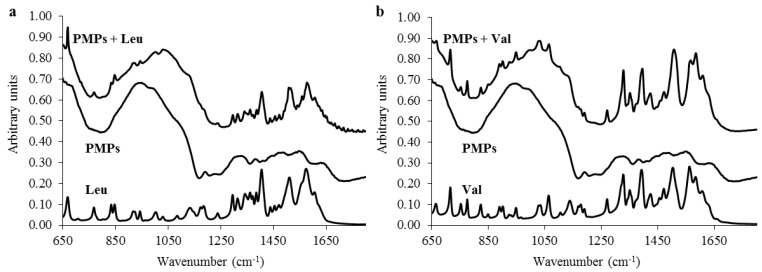
FT-IR spectra showing (**a**) leu, bare PMPs, and PMPs with bound leu, respectively; and (**b**) illustrates the FT-IR spectra of val, bare PMPs, and PMPs with bound val. Spectra were recorded from 4000 to 650 cm^−1^ at a resolution of 4 cm^−1^. Each spectrum was acquired by adding together 32 interferograms acquired at 22 °C.

To further elucidate the nature of interactions between PMPs and selected BCAAs, we employed attenuated total reflectance Fourier transform infrared spectroscopy (ATR-FT-IR). The FT-IR spectra of amino acids, bare PMPs, and amino acids bound on PMPs are shown in Figure 2a for leu and Figure 2b for val. From the spectra, it is clearly evident that there are no observable shifts in the wavenumbers of individual bands. Hence, it can be stated that the interactions between the amino acids and PMPs are of non-covalent nature and, thus, the binding between PMPs and amino acids does not result in the changes in biochemical and functional properties of bound BCAAs.

### 2.2. Encapsulation of Paramagnetic Nanoparticles into Liposome Inner Cavity

**Figure 3:**
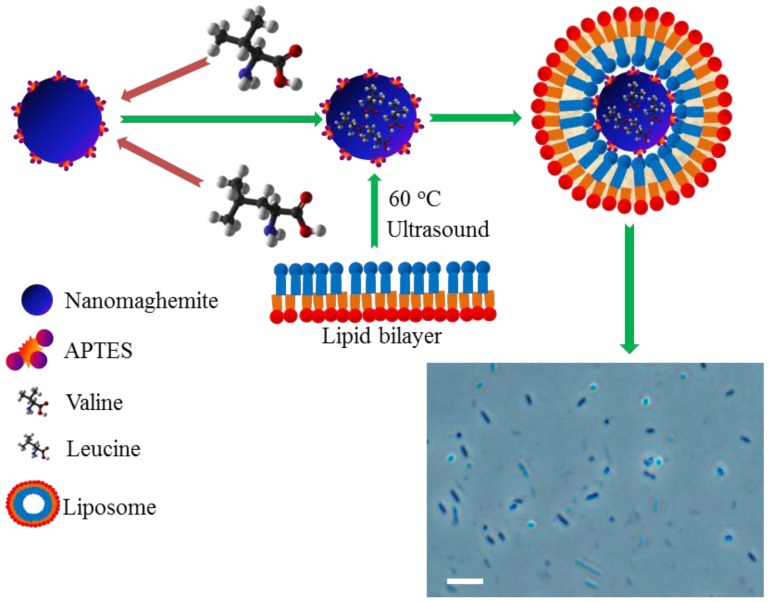
Scheme of the transporter complex with amino acids bound. BCAAs (val, leu) were bound onto the surface of the paramagnetic particles. Further, the paramagnetic complex was enclosed in a liposome to reduce disintegration of PMPs caused by undesired interactions with biomolecules and to increase the particle´s zeta potential to enhance the interactions with negatively-charged cells. The length of scale bar used in *E. coli* microphotography is 5 µM.

We decided to synthesize sterically-stabilized cationic liposomes, containing paramagnetic nanoparticles bearing BCAAs with the aim of generating stable liposomal carriers equipped with a payload of nanoparticles. As it can be seen in Figure 3, the first step included the isolation of amino acids using conditions optimized previously [25]. Subsequently, the liposome encapsulation was carried out. Due to the physicochemical properties of liposomes, controlled by the stability of sub-micron structures and lipid bilayer interfaces, it is possible to achieve prolonged persistence of the liposome cargo in the body and reduce its undesired degradation [26,27].

### 2.3. Evaluation of Transporter Complex Individual Parts Binding Affinity towards BCAAs

**Figure 4:**
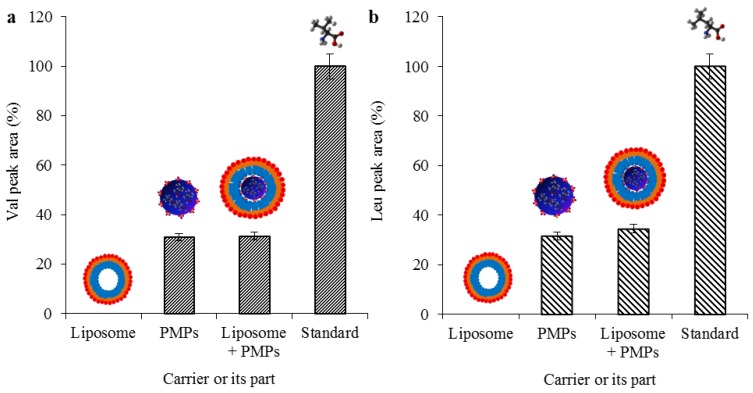
Expression of recoveries of (**a**) val; and (**b**) leu bounded to each part of the complex with a comparison with their standards. Concentrations of val and leu used for experiments was 100 µg·mL^−1^.

As a confirmation that the amino acids are bound only on the surface of PMPs, we have tested each individual part of the transporter complex on their binding affinity towards leu and val. As it is shown in Figure 4a,b, the amino acids were bound only when the paramagnetic particles were used (both with and without liposome) and the recoveries were more than 30% in all cases when compared with standards (31.3% for val bound on PMPs enclosed in liposome, and 32.6% for leu for PMPs enclosed in liposome). When the recoveries were converted to the initial concentrations, which were 100 μg·mL^−1^ for both amino acids, the resulting concentrations for val and leu were established to 31.3 and 32.6 μg·mL^−1^, respectively. The encapsulation resulted in a nanometric lipoplex-based complex (131 ± 24 to 142 ± 26 nm) with a zeta potential ranging from 28.876 ± 0.38 to 33.051 ± 0.54 mV, as it is shown in Table 1.

Concurrently with these findings, we obtained information about the affinity of individual parts of the complex towards leu and val. As can be seen in Figure 4, no amino acids were determined after incubation with empty liposomes. Thus, it was confirmed that the affinity of the amino acids is not influenced by some of the parts forming the entire nanocomplex and is fundamentally dependent on the presence of paramagnetic nanoparticles.

### 2.4. Uptake Efficiency of Lipoplex-Based Magnetic Nanotransporter Tested on E. Coli Strain

**Figure 5:**
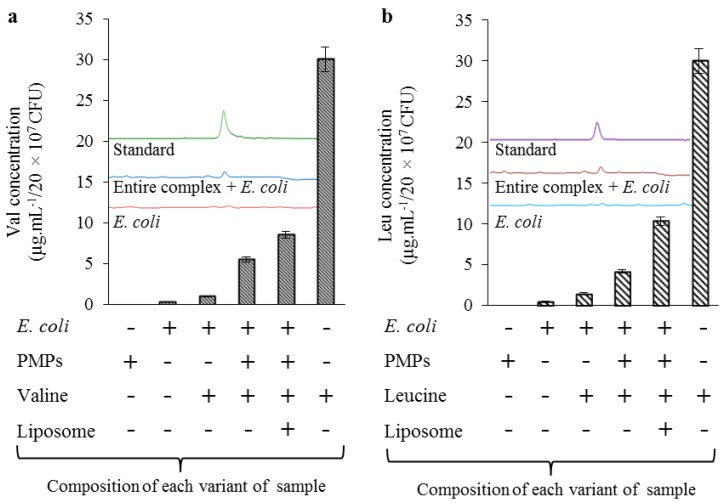
Expression of (**a**) valine; and (**b**) leucine uptake efficiency using an *E. coli* strain. Concentrations of amino acids used for treatment of *E. coli* were calculated at 30 µg·mL^−1^ in all cases and uptake efficiency is expressed as a concentration of amino acid (µg·mL^−1^) determined as 20 × 10^7^ CFU. Moreover, the chromatograms are showing the retention times of the amino acids of *E. coli* before and after the application of the entire complex, along with the standards.

We hypothesized that the overturn of negative zeta potentials to positive values, caused by a cationic liposome envelope, may enhance the interaction strength between the delivery system and negatively-charged cell membranes. For experiments, we decided to use *E.* *coli* NCTC 13216. Previously, it was explained that mostly all cells are negatively charged at neutral pH, due to the abundance of the anionic phosphatidylserine [28]. In *E. coli* strains, the exterior voltage may reach −50 mV [22]. Using IEC and high-performance liquid chromatography electrospray ionization quadrupole time-of-flight mass spectrometry (HPLC-ESI-QTOF MS), it was found that the encapsulation of BCAAs@PMPs into a liposomal envelope significantly enhanced the uptake of both valine and leucine in bacterial cells (Figure 5a,b).

As it can be seen in Table 2, the concentrations of BCAAs (in *E. coli*) after application of paramagnetic nanoparticles with no liposome envelope were found to be 5.5 and 4.1 µg·mL^−1^ for val and leu, respectively. After encapsulation, the uptake of BCAAs significantly increased to the values of 8.6 and 10.4 µg·mL^−1^ for val and leu, respectively. These results strongly suggest the applicability of a cationic liposomal encapsulation of various kinds of cargoes. Similar results were achieved by Lutwyche and coworkers, who have shown that liposomal encapsulation of aminoglycoside antibiotic gentamicin significantly enhances its uptake in the *Salmonella typhimurium* and *Listeria monocytogenes* strains [29]. Similarly, it has been demonstrated that a proper liposomal encapsulation of clarithromycin antibiotic improved its uptake in MRSA strain more than 25-fold [30]. Although we observed lower success rate of BCAAs uptake, our results are consistent with these findings and it should be noted that the rate of a non-encapsulated uptake is significantly influenced by a type of internalization. Independently, on a cargo, it has been shown that some specific types of liposomal vesicles, containing bromide or chloride in the lipid bilayer, can significantly affect the growth of bacterial cells by themselves [31].

Recalculations to uptake efficiency (%) described an enhancement of uptake between BCAAs@PMPs and BCAAs@PMPs in liposomes of about 10.4% in the case of val, and even 21.0% in the case of leu (Table 2). The determination of repeatability (five measurements) of BCAAs in cells after a single treatment was in the range 5.8%–8.5% and the reproducibility of five treatments ranged from 4.1% to 11.2%. Hence, it can be stated that the interactions between our designated magnetic-lipoplex system and *E. coli* cells demonstrate relatively constant trends and, thus, it is applicable for further experimental studies with the therapeutic antimicrobial peptides (AMPs).

### 2.5. Testing of Magnetic Lipoplex System Bearing Antimicrobial Peptide 

The important pleiotropic actions of AMPs, the many examples of relevance in animal models, and their associations with human disorders, all point toward this class of molecules as a new target for therapy [32]. Hence, we utilized L- and R-rich antimicrobial peptide (FALALKALKKALKKLKKALKKAL) to determine its interactions with our designated nanoparticles. Table 3 illustrates that nanoparticles were able to immobilize 15.1 µg·mL^−1^ of peptide. Furthermore, it was found that binding of peptides slightly elevates the entire complex size distribution and zeta potential, when compared to binding of single amino acids. An increase of zeta potential to a more positive value is considered as beneficial because the cationic nature of the peptide enhances the specificity of their binding to bacterial cell membranes relative to human cell membranes, because the former contain a greater amount of anionic lipids on the outer leaflet [33].

**Figure 6:**
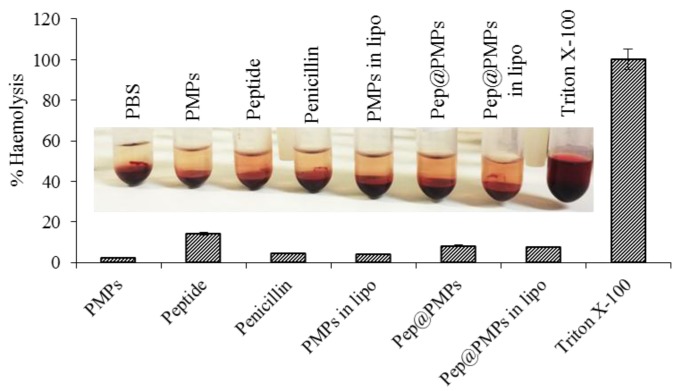
Hemocompatibility assay using human RBCs, showing hemolytic activity of the designated magnetic lipoplex system and its parts. Inserted is real photography after incubation and centrifugation. As a negative control, phosphate buffered saline (PBS) (pH 7.4) with no hemolytic activity was employed. As a positive control, 0.1% Triton X-100 was utilized. Absorbance of supernatant was determined at λ = 540 nm. Peptide and penicillin concentrations were 15 µg·mL^−1^ in all cases. Peptide concentrations were 15 µg·mL^−1^ in all cases.

Although we have obtained basic information about the peptide-bearing magnetic lipoplex system, further biological tests were required. Hemolytic assay was carried out to study the hemocompatibility or the lysing effect of the nanotransporter. Red blood cells (RBCs) are an important component of the blood circulatory system. The pivotal role of these cells is the transportation of oxygen from lungs to different tissues in the body. The hemolytic activity of peptides is a crucial parameter to estimate their therapeutic index. Strong membrane binding and insertion ability of peptides to bacteria could also threaten RBCs via similar membrane-disruption mechanisms. Thus, it is reasonable to tune the peptides to decrease these undesired side effects. Figure 6 demonstrates that, although individual peptides exhibited hemolytic activity close to 15%, its binding with nanoparticles and subsequent encapsulation can decrease this phenomenon (up to 8.2% in the case of pep@PMPs, or 7.5% in the case of pep@PMPs in lipo, respectively). Hence, it can be stated that encapsulation can enhance the therapeutic index of antimicrobial peptides due to protection of its payload against the external environment and the increase of circulation time.

**Figure 7:**
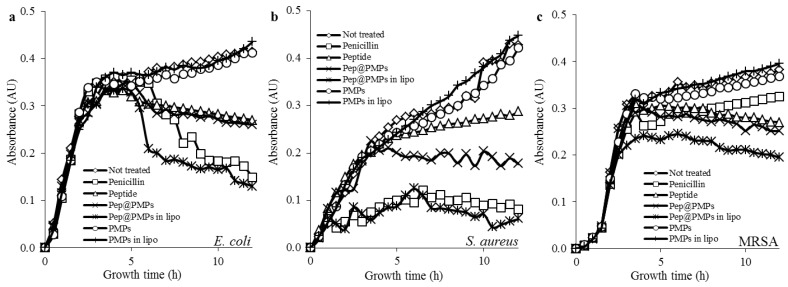
Growth curves of (**a**) *E. coli*; (**b**) *S. aureus*; and (**c**) MRSA treated with various parts of the magnetic lipoplex system bearing antimicrobial peptide or without peptide. Untreated bacterial cultures and cultures treated with conventional penicillin (15 µg·mL^−1^) were used as controls.

Finally, to determine the antimicrobial activity of the magnetic lipoplex delivery system we have tested its influence on the growth of selected bacterial strains (*E. coli*, *S. aureus*, and MRSA). As a positive control we chose conventional penicillin antibiotics, since the susceptibility of selected strains towards this substance is well documented [19]. Figure 7a,b illustrates that in both penicillin-susceptible bacterial strains (*E. coli* and *S. aureus*) the magnetic lipoplex nanotransporter caused similar growth inhibition effects as penicillin. Interestingly, individual peptide administration did not result in similar antimicrobial effects. In the case of *S. aureus*, treatment with pep@PMPs boosted antimicrobial efficiency, when compared to naked peptides. Nevertheless, the effects were much weaker than in the case of liposomal encapsulation. These data underscore the important role of the cationic envelope. The last tested bacterial strain was MRSA, which is well known for its resistance against beta-lactam antibiotics [34]. Contrary to other tested strains, MRSA was found to be significantly more susceptible to the magnetic lipoplex system (Figure 7c).

Based on obtained data, it is obvious that the cargo transmission may be enhanced significantly due to the electrostatic interactions between the target molecule and delivery system. This finding may be applied in the field of nanomedicine, where the more effective ways of targeted delivery are constantly researched. Nanomaghemite in complex offers both a presence as a contrast agent for MRI and/or as a photosensitizer for photodynamic therapy [35,36,37]. Moreover, a cationic lipoplex system may be helpful to improve the interactions of peptide-modified metal nanoparticles with bacterial membranes in peptide nanoparticles-based bacterial infection treatment [18], which represents a potential alternative of antibiotics, to which a larger resistance is still observed.

## 3. Materials and Methods

### 3.1. Materials

All of the reagents for synthesis, native oligonucleotides, BCAAs standard, and others, were purchased from Sigma Aldrich (St. Louis, MO, USA) in ACS purity, unless noted otherwise.

### 3.2. Synthesis of Nanomaghemite and Peptide

Nanomaghemite was prepared as follows: 1 g of FeCl_3_·6H_2_O was solubilized in 80 mL of MilliQ water and mixed subsequently with a solution of sodium borohydride (0.2 g in 10 mL ammonia, 3.5%). The obtained solution was heated at boiling temperature for 2 h. After cooling and keeping at room temperature for 2 h, the obtained superparamagnetic nanoparticles were separated by an external magnetic field using permanent magnets (Chemagen, Baesweiler, Germany) and washed several times with water. The resulting suspension was used for further modification.

The antimicrobial peptide with sequence FALALKALKKALKKLKKALKKAL (2537.32 Da, calculated net charge at pH 7 = 9) was prepared on Liberty Blue peptide synthesized (CEM, Matthews, NC, USA) by standard Fmoc solid-phase synthesis, using 20% piperidine in dimethylformamide (*v/v*) for deprotection. The purity of crude peptide was analyzed using HPLC-ESI-QTOF.

### 3.3. Modification of Nanomaghemite Core with APTES

20 mL of nanomaghemite suspension in water in a concentration of 1 mg·mL^−1^ was mixed with 2 mL of APTES in a concentration of 1 mg·mL^−1^. The resulting product was stirred on a Biosan OS-10 (Biosan, Riga, Latvia) overnight. Finally, the mixture was separated using the force of an external magnetic field (Chemagen, Baesweiler, Germany), washed three times with water, and dried at 40 °C.

### 3.4. Characterization of Nanomahemite@APTES Particles Morphology

The structure of paramagnetic particles was characterized by scanning electron microscopy MIRA3 LMU (Tescan, a.s., Brno, Czech Republic) was used. The model was equipped with a high-brightness Schottky field emitter for low noise imaging at fast scanning rates. An accelerating voltage of 15 kV and a beam current of about 1 nA were used to get satisfactory results regarding maximum throughput.

### 3.5. Determination of Size Distribution and Zeta Potential

Particle size and zeta potential of Nanomaghemite@APTES@ conjugate were evaluated using a particle size analyzer (Zetasizer Nano ZS90, Malvern instruments, Malvern, UK) as well as the size distribution and zeta potential of the entire complex. The conjugates were prepared in PBS (pH 7.4) and incubated at 25 °C for 15 min before measurement.

### 3.6. X-Ray Fluorescence Analysis of Paramagnetic Particles

X-ray fluorescence (XRF) element analysis of PMPs was carried out on Xepos (SPECTRO analytical instruments GmbH, Kleve, Germany). The parameters for analyses were as follows: measurement duration: 300 s, tube voltage from 24.81 to 47.72 kV, tube current from 0.55 to 1.0 mA, with zero peak at 5000 cps and vacuum switched off.

### 3.7. Workflow Process of Isolation of Amino Acids/Peptide Using APTES@Nanomaghemite

40 mg of PMPs was dissolved in 1000 µL of PBS to prepare stock solution. The stock solution was subsequently treated with an ultrasonic homogenizer SONOPULS mini20 (Bandelin electronic, Berlin, Germany) for 2 min to reduce the aggregation, evolved during the storage of particles in a dried state. 50 µL of suspension was further mixed with 200 µL of PBS. To remove the undesired impurities, nanoparticles were washed with PBS and Britton-Robinson buffer (pH 2), using a permanent magnet (Chemagen, Baesweiler, Germany). Isolation of amino acids/peptide (100 µg·mL^−1^ in all cases, diluted in phosphate buffered saline—PBS) was carried out according to the conditions, which we optimized in a previous study [25]. After isolation, the solution was removed and the remaining conjugate with amino acids was dissolved in 3 M HCl. The dissolved particles were quantitatively transferred into a 96-well evaporation plate (Deepwell plate 97, Eppendorf AG, Hamburg, Germany) and evaporated using the nitrogen blow-down evaporator (Ultravap with spiral needles, Porvair Sciences, Leatherhead, UK). Prior to IEC analyses, particles were re-suspended with a sodium dilution buffer.

### 3.8. Determination of Relative Current Response of Particles/Liquid Interface

Identification of the relative current response before and after BCAA binding was performed using a scanning electrochemical microscope, Model 920D (CH instruments, Austin, TX, USA). Measurements were carried out with 10 μL of paramagnetic articles in concentration of 10 μg·mL^−1^ mixed in ACS water. The electrochemical microscope consisted of a 10 mm platinum disc probe electrode with a potential of 0.2 V. Another platinum disc electrode was used with an O-ring as the conducting substrate with a potential of 0.3 V. During scanning, the particles were attached to the substrate platinum electrode by magnetic force from a neodymium magnet. The liquid used in the analyses consisted of 5% ferrocene in methanol (*w/v*) mixed in ratio 1:1 with 0.05% KCl in water (*v/v*). Measurements were performed in a Teflon cell with volume of 1.5 mL according to the following parameters: amperometric mode, vertical scan area 40 μm × 40 μm, and scan rate of 30 μm·s^−1^.

### 3.9. Ion-Exchange Liquid Chromatography Analyses of PMPs Chemical Moieties Specifity

For analysis of amino acids/peptides, bound on PMPs, an IEC (Model AAA-400, Ingos, Prague, Czech Republic) with post column derivatization by ninhydrin and absorbance detector in visible light range (Vis) was used, according to our previous study [25]. Limits of detection (3 × S/N), were established at 0.1 and 0.15 μg·mL^−1^ in case of val and leu, respectively, according to calibration curves (y = 0.00143x + 0.6466, R^2^ = 0.9941) and (y = 0.0027x + 0.053; R^2^ = 0.9752).

### 3.10. ATR-FT-IR

FT-IR spectra were collected using a Nicolet iS10 FT-IR spectrometer with a diamond ATR attachment (Thermo Electron Inc., San Jose, CA, USA). A uniform and thin layer of the finely-divided sample was placed in direct contact with the diamond crystal of the ATR cell. IR spectra were recorded from 4000 to 650 cm^−1^ at a resolution of 4 cm^−1^. Each spectrum was acquired by adding together 32 interferograms. Spectra were acquired at room temperature (22 °C) for each sample. The OMNIC™ software (Thermo Scientific, Waltham, MA, USA) was used for IR spectra recording and JDXview v0.2 software (Norbert Haider, Department of Drug Synthesis, University of Vienna, Austria) was used for further spectra evaluation.

### 3.11. Preparation of Liposomes

Liposomes were prepared by following the lipidic thin-film hydration method [38]. 100 mg of cholesterol, 100 mg of 1,2-dioleoyl-sn-glycero-3-phospho-rac-(1-glycerol) sodium salt, and 100 mg of phosphatidylcholine were dissolved in 4.5 mL of chloroform (*w/v*). A lipid film was obtained by rotary evaporation of solvent and residual chloroform was blown out by nitrogen to remove the organic solvent until a thin film was formed.

### 3.12. Encapsulation of Particles Bearing BCAAs/Peptide into Liposome Inner Cavity

For liposome encapsulation, the solution, composed of 1 mg·mL^−1^ of paramagnetic particles (with 100 µg·mL^−1^ of BCAAs bound) in water was utilized. Upon mixing with a surplus (20 mg) of prepared liposomes, the samples were homogenized in an ultrasonic bath using a Sonorex Digital 10P (Bandelin, Berlin, Germany) for 15 min. The homogenized mixtures were then heated and shaken for 15 min at 60 °C using a Thermomixer Comfort (Eppendorf AG, Hamburg, Germany). The samples were then washed several times with Britton-Robinson buffer (pH = 10) on an Amicon 3k (Millipore, Billerica, MA, USA) to obtain the product with maximum purity.

### 3.13. Escherichia Coli, Staphylococcus Aureus, and MRSA Cultivation, Treatment, and Harvest

*E. coli* (NCTC 13216), *S. aureus* (NCTC 8511), and MRSA were obtained from the Czech Collection of Microorganisms, belonging to Faculty of Science, Masaryk University, Brno, Czech Republic. The cultivation medium consisted of 5 g·L^−1^ meat peptone, 5 g·L^−1^ NaCl, 1.5 g·L^−1^ bovine extract, 1.5 g·L^−1^ yeast extract (HIMEDIA, Mumbai, India) and sterilized MilliQ water with a resistance of 18 MΩ and pH adjusted to 7.4. The sterilization of the media was carried out at 121 °C for 30 min in sterilizer (Tuttnauer 2450EL, Bread, Netherlands). After inoculation, the bacterial cultures were cultivated for 24 h on a shaker at 600 rpm and 37 °C. The bacterial cultures, cultivated under these conditions was diluted by cultivation medium on a Specord spectrophotometer 210 (Analytic, Jena, Germany) to OD_600_ = 0.1 (3.7 × 107 CFU.mL^−1^) and used in the following experiments. The BCAA uptake experiments were carried out with 250 µL of *E. coli* mixed together with 50 µL of BCAAs (30 µg·mL^−1^). The interaction was carried out for 24 h at 37 °C and, subsequently, the cells were harvested and the magnetic liposomes were removed with an external magnetic field (Chemagen, Baesweiler, Germany). After that, the solutions of treated *E. coli* were centrifuged using a Microcentrifuge 5417R (Eppendorf AG, Hamburg, Germany) in conditions as follows: 6000 rpm, 4 °C for 30 min. Resulting pellets were hydrolyzed in 3 M HCl using a microwave reactor Anton Paar (Anton Paar GmbH, Graz, Austria), in the following conditions: 90 min, 120 °C, pressure 25 bar. The sample was diluted 10 times with dilution buffer composed of thiodiglycol (5 mL·L^−1^), citric acid (14 g·L^−1^), and sodium chloride (11.5 g·mL^−1^). The resulting samples were analyzed using ion-exchange chromatography in the conditions mentioned above.

### 3.14. HPLC-ESI QTOF Mass Spectrometry

The level of amino acid uptake was evaluated using HPLC-ESI-QTOF. For HILIC separation, the chromatographic column Luna HILIC 200A (150 × 4.6; 3.5 µm particles, Phenomenex, Torrance, CA, USA) was used according to our previous study [39]. 0.1% of formic acid (*v/v*) was added in to the polar part of the mobile phase to enhance the ionization. For detection, a Bruker Maxis Impact mass spectrometer with electrospray by ionization quadrupole time-of-flight (Bruker, Munich, Germany) was utilized. The electrospray ionization source was operated in a positive mode. The voltage of the electrospray capillary was set to 3500 V with a nebulizing gas (N_2_) flow rate of 4 L·min^−1^ and a drying gas temperature set to 350 °C. Scanning was carried out within the range from 50 to 1000 *m/z*. The samples were diluted 1000 times in isopropanol prior to analysis.

### 3.15. Hemocompatibility Assay

Hemolytic assay was carried out to check the hemocompatibility of the designated nanotransporter on human erythrocytes. Fresh blood sample was centrifuged (2000 rpm, 5 min) to remove plasma and serum. Then, different parts of the nanotransporter in PBS (pH 7.4) were mixed with the red blood cells (RBCs) and incubated for 1 h at 37 °C. PBS and 0.1% Triton X-100 were used as negative and positive controls, respectively. After completion of the incubation period, the cells were centrifuged and the absorbance of the supernatant containing lysed erythrocytes was measured at 540 nm. The percentage of hemolysis was determined by the following equation: %hemolysis = ((At − Ac)/A100% − Ac)) × 100, where At is the absorbance of the supernatant from samples incubated with the particles; Ac is the absorbance of the supernatant from negative control; and A100% is the absorbance of the supernatant of positive control (0.1% Triton X−100), which causes complete lysis of RBCs.

### 3.16. Determination of Growth Curves

Growth curve measurements were carried out using a Multiskan EX (Thermo Fisher Scientific, Bremen, Germany). *E. coli*, *S. aureus*, and MRSA prepared as is described in Section 3.13 was pipetted into a microplate alone as a control, or with various parts of the magnetic lipoplex system. The concentration of peptide and penicillin was always 15 µg·mL^−1^. Measurements were carried out at time 0, then each half-hour for 24 h at 37 °C, at a wavelength of 620 nm.

### 3.17 Descriptive Statistics

Mathematical analyses of the experimental data and their graphical interpretation were carried out using the Microsoft Office tools (MS Excel^®^, MS Word^®^, and MS PowerPoint^®^, version 2010). All results were expressed as a mean ± standard deviation (SD) unless noted otherwise.

## 4. Conclusions 

In summary, we showed the synthesis of paramagnetic nanoparticles composed of nanomaghemite cores and APTES shells, and how the encapsulation in cationic liposome cavities influenced their zeta potential and delivery efficiency, tested in *E. coli* bacterial cells. The functionalized nanometric maghemite exhibits affinity towards BCAAs where, in particular, val and leu were bound with the highest recoveries. Binding affinity was employed to propose a delivery system of amino acids, composed of BCAAs bound on the surface of modified nanomaghemite encapsulated in cationic liposomes. The entire complex was shown to be positively charged and, thus, willingly interacts with cell membranes (mostly having negative exterior voltage) through electrostatic interactions. From the analyses of uptake efficiency, we can conclude that the encapsulation leading to the overturn of zeta potential may be applicable for improvement of effective delivery systems. Moreover, it was demonstrated that the cationic lipoplex systems represents a potential alternative method of the treatment of infections, based on cationic peptides, whose treatment efficacy can be improved; however, further *in vivo* experiments might be performed to determine the real effect of advanced nanomaterials on the peptides’ therapeutic index. 

## Figures and Tables

**Table 1:** Expression of basic characteristics of the entire lipoplex-based nanotransporter.

Cargo	Concentration Used for Encapsulation	Concentration Determined (Recovery)	Complex Size Distribution	Complex Zeta Potential
Unit	µg·mL^−1^	µg·mL^−1^	nm	mV
Val	100	31.3	131 ± 24	28.88 ± 0.38
Leu	100	32.6	142 ± 26	33.05 ± 0.54

**Table 2:** Expression of BCAAs uptake efficiency, determined in an *E. coli* strain, and standardized to 20 × 10^7^ CFU, according to growth curves.

Treatment Agent	Concentration Applied	Concentration Analyzed in E. Coli *	Repeatability ** (*n* = 5)	Uptake Efficiency	Reproducibility *** (*n* = 5)
Unit	µg·mL^−1^	µg·mL^−1^	(%)	(%)	(%)
Val	30	1.0	5.8	3.3	9.8
Val@PMPs *	30	5.5	8.5	18.3	10.0
Val@PMPs in lipo *	30	8.6	7.1	28.7	8.2
Leu	30	1.5	6.2	5.0	4.1
Leu@PMPs *	30	4.1	6.9	13.7	11.2
Leu@PMPs in lipo *	30	10.4	7.6	34.7	6.1

* Calculated from recovery, obtained from previous experiments. ** Relative standard deviations of determined repeatability after one treatment (*n* = 5). *** RSDs of reproducibility of five independent treatments.

**Table 3:** Expression of basic characteristics of entire lipoplex-based nanotransporter with immobilized antimicrobial peptide.

Cargo	Concentration Used for Encapsulation	Concentration Determined (Recovery)	Complex Size Distribution	Complex zeta Potential
Unit	µg·mL^−1^	µg·mL^−1^	nm	mV
Peptide	50	15.1	168 ± 49	39.11 ± 4.01

## Abbreviations

The following abbreviations are used in this manuscript:

BCAAs: Branched chain amino acids
Val: Valine
Leu: Leucine
Ile: Isoleucine
Arg: Arginine
APTES: (3-aminopropyl)triethoxysilane
mTOR: mammalian target of rapamycin
MRI: Magnetic resonance imaging
*E. coli*: *Escherichia coli*
*S. aureus*: *Staphylococcus aureus*
MRSA: Methicilin-resistant *S. aureus*
HPLC-ESI-QTOF: High performance liquid chromatography electrospray ionization quadrupole time of flight
RBCs: Red blood cells
SEM: Scanning electron microscopy
IEC: Ion-exchange chromatography
SECM: Scanning electrochemical microscope
ATR-FT-IR: Attenuated total reflectance Fourier transform infrared spectroscopy
PMPs: Paramagnetic particles
AMPs: Antimicrobial peptides
PBS: Phosphate buffered saline
XRF: X-ray fluorescence
